# A multi-level fuzzy comprehensive evaluation model to optimize biochar application schemes for potato cultivation in North China

**DOI:** 10.3389/fpls.2025.1571305

**Published:** 2025-06-05

**Authors:** Jiawei Guo, Hui Zhou, Liguo Jia, Yongqiang Wang, Mingshou Fan, Meirong Wang, Peng Liu, Zhihui Shang

**Affiliations:** ^1^ College of Agronomy, Inner Mongolia Agricultural University, Hohhot, China; ^2^ Yinshanbeilu Grassland Eco-Hydrology National Observation and Research Station, China Institute of Water Resources and Hydropower Research, Beijing, China; ^3^ Institute of Water Resources for Pastoral Area Ministry of Water Resources, Hohhot, China; ^4^ Ulanqab City Inspection and Testing Center, Ulanqab, China

**Keywords:** biochar, pyrolysis temperature, potato, optimization, soil nutrient residues, comprehensive model

## Abstract

**Introduction:**

The North China region is a major potato production area, but water scarcity and poor soil fertility limit potato growth. Biochar is a promising approach to improve soil quality and enhance crop productivity. However, the effects of different biochar pyrolysis temperatures and application rates on potato growth, economic benefits, quality, water and fertilizer use efficiency, and soil nutrient retention remain unclear.

**Methods:**

A field experiment was conducted during 2023–2024 to evaluate the effects of biochar pyrolysis temperatures (T1: 300°C, T2: 500°C, T3: 700°C) and application rates (C1: 10 t ha^−1^, C2: 20 t ha^−1^, C3: 30 t ha^−1^) on comprehensive potato cultivation performance. A multi-level fuzzy comprehensive evaluation (PFCE) model was used to determine the optimal biochar application strategy.

**Results:**

Potato growth indicators, water and fertilizer use efficiency, starch, and vitamin C content exhibited a parabolic trend, with the C2T2 treatment performing best. Net income was highest for the CK treatment in 2023 and for C2T2 in 2024. Reducing sugar content was lowest in C2T2; soil nitrate nitrogen accumulation was lowest in C3T2; soil available phosphorus was lowest in C1T3; and soil available potassium was lowest in CK. PFCE analysis indicated that C2T2 achieved multi-objective optimization for yield, quality, efficiency, and environmental sustainability.

**Discussion:**

Based on PFCE results and practical production considerations, applying biochar at 400–500°C pyrolysis temperature and 18–20 t ha^−1^ application rate is recommended for North China to maximize comprehensive benefits.

## 1 Introduction

Globally, potatoes are one of the most essential non-cereal and high-yield horticultural crops, with an annual tuber production reaching 370 million tons (Canton, 2021). Potatoes are a nutritionally rich food and vegetable that play a significant role in providing nutritious food, ensuring food security, and promoting agricultural development. In North China’s arid and semi-arid regions, potatoes are an essential staple crop; however, water scarcity and poor soil fertility are critical factors limiting potato growth and yield (Wu et al., 2022). Local farmers often excessively use irrigation water and fertilizers in pursuit of high potato yields, resulted in decreasing water and nutrient use efficiency and soil structure degradation, ultimately reducing crop yield and quality. Therefore, optimizing agricultural production technologies to improve water and nitrogen use efficiency in North China’s arid and semi-arid regions is crucial for ensuring sustainable farmland utilization and crop production.

Biochar has been evaluated for its role in soil improvement in recent years. Numerous studies conducted through greenhouse experiments, pot trials, or field studies have assessed the effects of biochar on soil properties, plant growth, yield, and quality, while also exploring its potential to enhance economic and environmental benefits (Faloye et al., 2019; Han et al., 2023; Wang et al., 2023). Despite variations in results across different studies, a positive impact of biochar application on agricultural production is generally observed. Research by Jia et al. (2021) indicates that biochar has beneficial effects on improving soil health and increasing water and nutrient use efficiency. This is mainly attributed to its porous structure and high specific surface area, which enhance soil moisture and nutrients retention capacity, and reduce nitrogen leaching losses and ammonia volatilization (Alkharabsheh et al., 2021; Dey et al., 2023). Additionally, hydroxyl and carboxyl groups in biochar increase soil adhesion and cohesion, improving nutrient availability and promoting better plant growth and higher yields (Yu et al., 2019). The application rate of biochar is a crucial factor affecting crop productivity (Abukari et al., 2022; Gao et al., 2021; Singh et al., 2022). Multiple studies have shown that biochar, as a soil amendment, often requires substantial application rates, such as 10 to 50 t ha^-1^ (Dong et al., 2022; Yan et al., 2022; Medyńska-Juraszek et al., 2021), with some suggested optimal amount as high as 135.2 t ha^-1^ (Lehmann et al., 2003). However, other research indicates that excessive biochar application (≥20 t ha^-1^) can inhibit crop growth, reduce soil microbial activity, and hinder nutrient uptake (Gao et al., 2019; Liu et al., 2022). Moreover, the economic feasibility of high biochar application rates for farmers is also an important consideration. Currently, there is limited research on the effects of biochar on potato growth, tuber yield, water and nutrient use efficiency, and quality. For example, Mollick (2018) found that applying 7.5 t ha^-1^ of biochar can promote potato growth and achieve high yields. Wang et al. (2023) conducted pot experiments that demonstrated that the application of 7% biochar improves soil enzyme activity, increases plant chlorophyll and antioxidant content, ultimately enhancing potato quality and yield. Youssef et al. (2017) studied the effects of applying 5 t ha^-1^ of biochar under sandy soil conditions, concluding that it can increase net returns from potato production and reduce environmental pollution. It is worth noting that current research mainly focuses on the impact of biochar application rates on potato productivity. However, the effects of biochar’s functions and properties on soil and crops should not be overlooked (Ayaz et al., 2021; Brtnicky et al., 2021). Particularly under varying biochar application rates, the influence of biochar properties on the comprehensive aspects of potato growth, economics, quality, water and nutrient use, and environmental benefits in North China remains to be clarified.

The properties and functions of biochar are determined by its preparation processes and the types of raw materials used. Among these, pyrolysis temperature is one of the main factors influencing biochar properties (Janu et al., 2021; Ippolito et al., 2020; Hassan et al., 2020). The composition and forms of nutrients in biochar change with the variations in pyrolysis temperature (Guo et al., 2021). For instance, the specific surface area, porosity, pH, ash content, and carbon content of biochar increase with rising pyrolysis temperatures. In contrast, the O/C ratio and volatile matter content exhibit the opposite trend (Das et al., 2021). Changes in biochar properties and functions due to different pyrolysis temperatures can directly or indirectly affect crop growth. Li et al. (2019) found that biochar produced at temperatures of 400–500°C is most effective for increasing crop yields, whereas biochar produced at higher temperatures (especially >600°C) can reduce crop yields. Purakayastha et al. (2019) also concluded that exceeding a specific pyrolysis temperature range can diminish a crop’s ability to absorb soil nutrients, leading to reduced yields. However, some studies indicate that biochar produced at high pyrolysis temperatures (550°C and above) can be a desirable soil amendment due to its unique functional groups, which exhibit low toxicity to soil organisms and promote soil health and ecological balance. As a result, high-temperature biochar may improve the growth environment for crops better than low-temperature biochar (Tang et al., 2020). Therefore, to ensure sustainable agricultural practices, it is essential to consider the interactive effects of biochar pyrolysis temperature and application rate on potato growth, economics, quality, water and nutrient use, and environmental benefits. This will help identify the optimal biochar application strategy for potato fields in North China’s arid and semi-arid regions.

Multi-objective comprehensive evaluation methods effectively assess the strengths and weaknesses of various schemes, leading to reliable and reasonable evaluation results (Wang et al., 2019). The Analytic Hierarchy Process (AHP), which combines qualitative and quantitative analysis, is an effective decision-making tool for calculating the weights of evaluation indicators (Panchal and Shrivastava, 2022). However, the weight calculations in AHP are highly dependent on personal judgment, which can be influenced by biases and experiences, resulting in a lack of precision. In contrast, the Entropy Method (EP) minimizes the impact of subjectivity on weights, making the evaluation results more reflective of objective realities (Wang et al., 2021). Nevertheless, EP requires that input data be quantitative and has limited capability for handling qualitative indicators. Combining AHP and EP weighting methods can harness the advantages of both approaches, overcoming the limitations of individual techniques and enhancing the scientific and rational basis for decision-making (Xiao et al., 2021). Fuzzy Comprehensive Evaluation (FCE), based on fuzzy mathematics, transforms qualitative assessments into quantitative evaluations through membership theory. Determining indicator weights is fundamental to its methodology, directly impacting the reasonableness and reliability of evaluation results (Han et al., 2020). Thus, the multi-level fuzzy comprehensive evaluation optimization model (PFCE) framework integrates qualitative and quantitative data to improve evaluation accuracy, yielding broader and more accurate results. Furthermore, existing studies mainly select the best scheme based on evaluation results without further modeling analysis, often leading to inaccuracies (Wang et al., 2023). Therefore, the objectives of this study are: (1) to clarify the interactive effects of biochar pyrolysis temperature and application rate on potato dry matter accumulation, tuber yield, quality, water and nutrient use efficiency, soil nutrient retention and economics, through two years of field trials; (2) to establish a comprehensive evaluation system for the quality, water and nutrient use efficiency, environmental benefits, and economics of potatoes using the multi-level fuzzy comprehensive evaluation model (PFCE); (3) to identify the optimal biochar pyrolysis temperature and application rate for maximizing comprehensive benefits in potato fields in the arid and semi-arid regions of North China.

## 2 Materials and methods

### 2.1 Experimental site

Field experiments were conducted from 2023 to 2024 at the Kobuer Experimental Station in Chayouzhongqi, Ulanqab City, Inner Mongolia Autonomous Region (Latitude: 41°17′59.81″N, Longitude: 122°33′26.65″E). The experimental area is in an arid and semi-arid region with a temperate continental climate characterized by an average frost-free period of approximately 100 days and an annual mean temperature of 1.4°C. The long-term average wind speed is 4.8 m s^-1^, and the long-term average evaporation rate is about 2000 mm, while the average yearly precipitation is approximately 300 mm. Within the experimental area, a field micro-meteorological station (HOBO-U30) was established to record meteorological data automatically. The temperature, precipitation, average wind speed, and relative humidity during the potato growing season in 2023–2024 are shown in **Supplementary Figure S1**. The soil texture in the 0~60 cm layer is sandy loam, while below 60 cm, it is loamy sand. The initial soil properties (0~100 cm) are presented in **Table 1**.

**Table 1:** Basic soil properties of the experimental area.

Soil layer (cm)	Bulk density (g cm^-^³)	pH	Organic matter (g kg^-1^)	Total nitrogen (g kg^-1^)	Available phosphorus (mg kg^-1^)	Available potassium (mg kg^-1^)
0~20	1.39	8	26.5	2.22	17.50	119.5
20~40	1.41	7.9	10.12	0.62	6.93	78.32
40~60	1.42	8.1	7.38	0.48	3.58	63.28
60~80	1.45	8	3.58	0.37	2.81	48.51
80~100	1.4	7.9	2.23	0.25	2.69	39.25

### 2.2 Experimental design

Biochar was purchased from Zhengzhou Haosen Environmental Protection Technology Co., Ltd., China. The preparation process was as follows: wheat straw was dried and crushed, then passed through a 200-mesh sieve before being placed in a rotary kiln. The temperature was increased at a rate of 10°C min^-1^ to 300°C, 500°C, and 700°C, respectively, and maintained for 0.5 hours. Based on the final temperature, the biochar types were classified as T1 (low temperature), T2 (medium temperature), and T3 (high temperature), respectively. The physicochemical properties of the biochar are detailed in **Supplementary Table S1**.

In 2023–2024, ten treatment schemes were established in the potato farmland, including one control group (CK) and interaction treatments of biochar at three different pyrolysis temperatures (300°C, 500°C, and 700°C) and three application rates (10 t ha^-1^, 20 t ha^-1^, and 30 t ha^-1^) (see **Supplementary Table S2** for specific experimental design). Before planting in 2023, biochar was evenly spread over the soil surface of each plot, followed by thorough mixing with the top 0~20 cm of soil using a rotary tiller. Each plot measured 4 m × 5 m and was replicated three times. A completely randomized design was employed to arrange the positions of different plots, with a 1.5 m wide buffer zone established between adjacent plots to avoid cross-contamination during irrigation and fertilization.

### 2.3 Experimental layout

**Figure 1:**
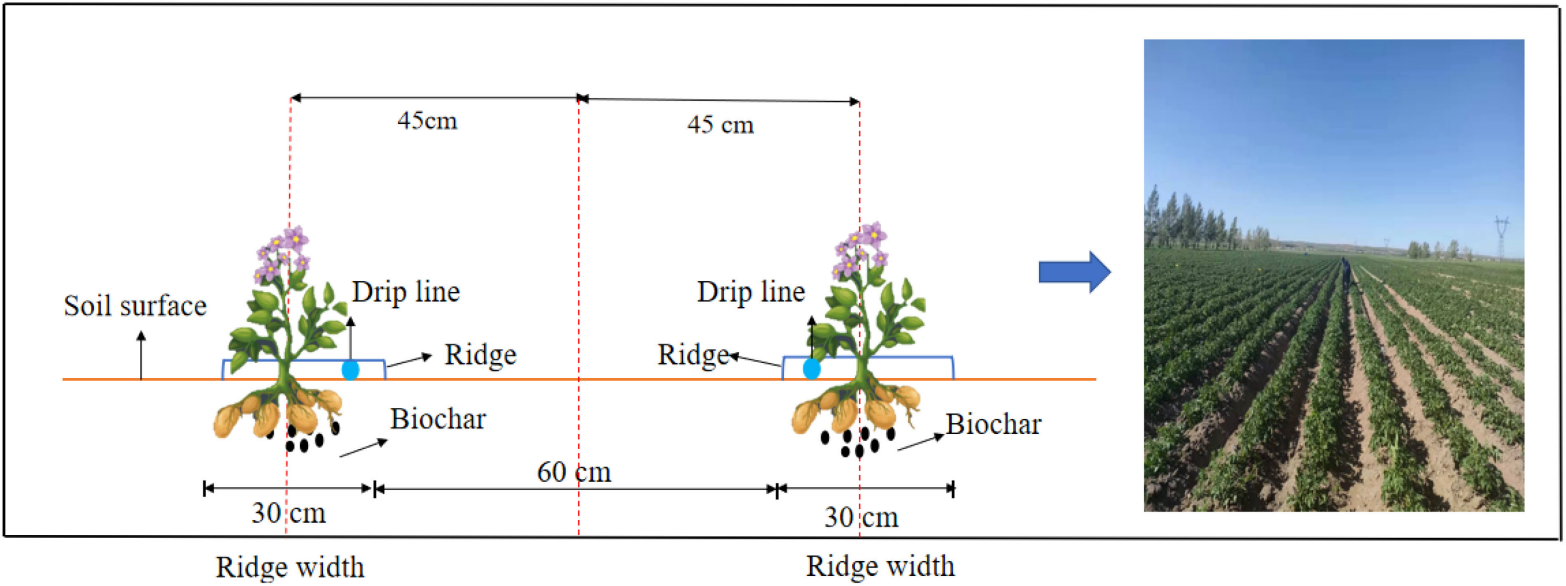
Experimental layout diagram.

The potato variety used in the experiment was Jinshu 16, with planting dates of May 9 in both 2023 and 2024, and harvest dates of September 13 and 14, respectively. A ridge cultivation mode was adopted, with a row spacing of 90 cm, ridge height of 20 cm, plant spacing of 28 cm, ridge length of 23 m, and ridge width of 30 cm, as detailed in **Figure 1**. The irrigation method employed was drip irrigation, using drip tape with a wall thickness of 0.18 mm, a drip emitter spacing of 0.3 m, an emitter flow rate of 2.2 L h^-1^, and an operating pressure of 0.1 MPa. The irrigation and fertilization regime was developed based on local farmers’ practices, with specific irrigation amounts outlined in **Supplementary Table S3**. Fertilization utilized commonly used local fertilizers, with urea (N: 46%) applied at a rate of 652.2 kg ha^-1^, where 30% was broadcast at sowing, followed by top dressing of 30% of the total nitrogen during the tuber formation period and 40% during the bulking period. Calcium superphosphate (P_2_O_5_: 46%, application rate of 391.3 kg ha^-1^) and potassium sulfate (K_2_O: 45%, application rate of 666.6 kg ha^-1^) were used as base fertilizers, applied to the soil using a combined fertilization and planting machine.

### 2.4 Measurements and calculation

#### 2.4.1 Growth, yield, and nutrient accumulation of potatoes

Three representative potato plants were randomly sampled from each experimental plot to measure the height, leaf area, dry matter weight, and nitrogen, phosphorus, and potassium accumulation at maturity. The specific methods are as follows:

Plant Height: Measured using a measuring stick with an accuracy of 0.1 cm.

Leaf Area Index (LAI): The leaf area was measured using a hole punch (10 cm diameter), and the LAI was calculated as the ratio of total leaf area to the area of land occupied (Watson, 1947) (See details in Equation 1).


(1) $$\text{LAI}=\frac{\text{Total Leaf Area​}}{\text{Ground Area}}$$

Dry Matter Weight: The plant samples were decomposed and cleaned, and surface moisture was removed using absorbent paper. The samples were blanched at 105°C for 30 minutes and then dried at 75°C until a constant weight was reached. The dry matter weights of the leaves, stems, roots, and tubers were measured with an accuracy of 0.01 g (Wang et al., 2021).

Chlorophyll Content: Eighty days after planting, ten representative potato plants from each plot were selected, and the relative chlorophyll content in the leaves was measured using a handheld chlorophyll meter (Yan et al., 2021).

Nitrogen, Phosphorus, and Potassium Accumulation in Different Organs: Each organ’s dry matter was ground, passed through a 0.5 mm sieve, and digested using H_2_SO_4_-H_2_O_2_. Total nitrogen was measured using a Kjeldahl nitrogen analyzer, total phosphorus was analyzed using a continuous flow analyzer, and total potassium was measured using atomic absorption spectrophotometry (Wang et al., 2021).

Potato Yield: At harvest, fresh potatoes from an area of 2 m (length) × 1.8 m (width) were weighed, with each plot being weighed three times. The tuber yield for each plot was calculated based on the weighing results and converted to a per-hectare basis (Wang et al., 2021).

#### 2.4.2 Net income and tuber quality

Net Income: Calculated as total income minus input costs (including seed, pesticide, water, electricity, chemical fertilizers, pipe network, and labor).

Tuber Quality: Three tubers were randomly selected from each plot for fresh sample measurement at harvest. Starch content was determined using the iodine colorimetric method, reducing sugar content was measured using the 3,5-dinitrosalicylic acid colorimetric method, and vitamin C content was determined using titration (Zhang et al., 2022).

#### 2.4.3 Potato water and fertilizer utilization efficiency

Water Use Efficiency (WUE) of Potato (Equation 2):


(2) $$WUE=\frac{\text{Y}}{10ET}$$

Where *WUE* represents Water Use Efficiency (kg m^-3^); *Y* denotes crop yield (kg ha^-1^); *ET* refers to crop water consumption (mm, Equation 3).


(3) ET=P+U+I−D−R−ΔW

Where *P* represents rainfall (mm); *U* denotes groundwater recharge (mm); *I* is the irrigation amount (mm); *R* indicates runoff (mm); *ΔW* refers to the change in soil moisture from the beginning to the end of the experiment (mm).

Given that the groundwater is deeply buried and the terrain is flat in the experimental area, both groundwater recharge and surface runoff are considered negligible. The change in soil moisture (ΔW) refers to the variation in soil water content within the 0–100 cm soil profile from sowing to harvest (Oweis et al., 2011).

The adequate rainfall *P_0_=aP*. When rainfall is less than 5 mm, a=0; when rainfall is between 5 mm and 50 mm, it ranges from 1.0 to 0.8; when rainfall exceeds 50 mm, it ranges from 0.70 to 0.80 (Guo, 1986). This can be simplified as Equation 4:


(4) $$ET=P_{0}+I-\Delta W-D$$

Where *P_0_* represents adequate rainfall (mm), *I* denote the irrigation amount (mm), *D* represents the amount of deep percolation (mm).

*ΔW* refers to the change in soil moisture from the beginning to the end of the experiment (mm).

Fertilizer partial factor productivity (Equation 5):


(5) $$PFP=\frac{Y}{FT}$$

Where *PFP* represents the fertilizer partial productivity (kg kg^-1^); *FT* denotes the total input of N, P_2_O_5_, and K_2_O (kg ha^-1^) (Cassman et al., 1996).

#### 2.4.4 Accumulation of nitrate nitrogen, available phosphorus, and available potassium

During the potato harvest periods in 2023 and 2024, soil samples were collected from three locations within each plot (furrow, below the drip head, and in the middle of the ridge) at a 100 cm depth divided into five layers. A 5 g soil sample was extracted with 50 mL of 2 mol L^-1^ potassium chloride solution for nitrate nitrogen determination. The mixture was shaken for 0.5 hours and then filtered. The nitrate nitrogen content in the soil was measured using an AA3 continuous flow analyzer (Bran+Luebbe, Germany) (Bao, 2000). To measure available phosphorus, a 5 g soil sample was extracted with 0.5 mol L^-1^ sodium bicarbonate solution (pH = 8.5). After shaking and filtering, the available phosphorus content in the soil was determined using the molybdenum-antimony anti-colorimetric method (Bao, 2000). For available potassium, a 5 g soil sample was extracted using 1 mol L^-1^ neutral ammonium acetate, and the available potassium content was determined using the flame photometry method (Bao, 2000).

The accumulation of soil nitrate nitrogen (or available phosphorus or available potassium) can be calculated as follows (Yan et al., 2021, Equation 6):


(6) $$C=\frac{M\cdot H\cdot B}{10}$$

Where *C* represents the accumulation of soil nitrate nitrogen (or available phosphorus or available potassium) (kg ha^-1^); *M* denotes the content of nitrate nitrogen (or available phosphorus or available potassium) in the soil (mg kg^-1^); *H* is the thickness of the soil layer (20 cm); and B is the soil bulk density (g cm^-3^).

### 2.5 Multi-level fuzzy comprehensive evaluation optimization model

#### 2.5.1 Determination of factor weights using analytic hierarchy process

(1) Establishing a Comprehensive Performance Evaluation Index System

**Figure 2:**
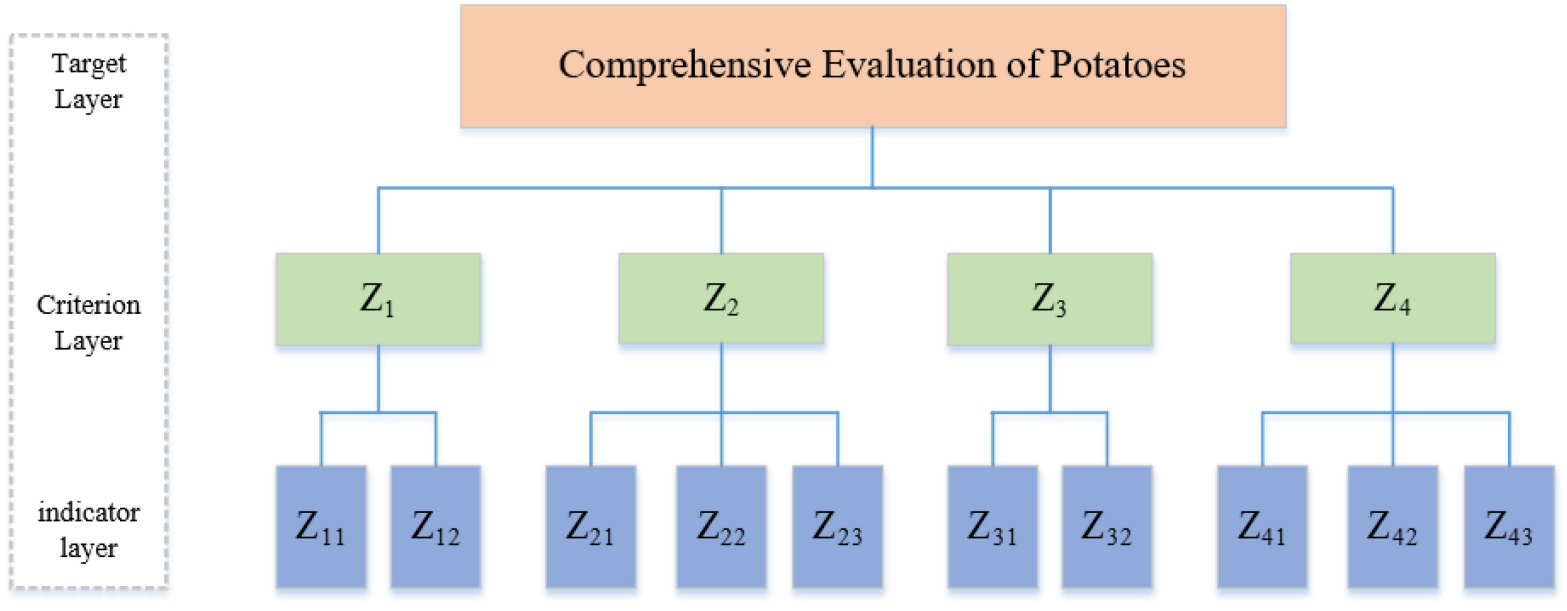
Comprehensive evaluation system for potatoes. Z_1_ is the economic benefit, Z_11_ is the potato Yield, Z_12_ is the net income, Z_2_ is the quality benefit, Z_21_ is the starch content, Z_22_ is the vitamin C content, and Z_23_ is the reducing sugar content. Z_3_ is water and fertilizer utilization efficiency, Z_31_ is water use efficiency, Z_32_ is fertilizer utilization efficiency; Z_4_ for environmental benefit, Z_41_, Z_42,_ and Z_43_ for soil nitrate nitrogen, available phosphorus, and available potassium residue, respectively.

The Analytic Hierarchy Process (AHP), proposed by Saaty in 1980, aims to determine the overall evaluation objectives based on the evaluation purpose. The comprehensive evaluation system is divided into three levels: the top level is the goal level, the second level consists of the criteria for evaluating the objectives, and the third level includes specific indicators (**Figure 2**). Regarding the relationships among various factors in potato cultivation, the goal level is set as the interaction effect of biochar pyrolysis temperature and application rate on potato Yield, economic benefits, tuber quality, water and fertilizer utilization efficiency, and environmental benefits. The criteria level includes economic benefits, quality, water and fertilizer utilization efficiency, and ecological benefits of potatoes. The indicators level comprises specific indicators for evaluating the factors, forming a judgment matrix reflecting the hierarchical relationships within the comprehensive system.

When constructing the matrix, five irrigation experts were interviewed using a questionnaire, and they compared the importance of several indicators based on Saaty’s 9-point scale (**Table 2**), forming the judgment matrix B(1−6). The subjective weights in constructing matrix A were calculated as follows:

**Table 2:** The grading standards “1-9” at various levels and their reciprocal scale method.

Scale value	Relative important degree	Illustration
1	Equally important	Both evaluation elements contribute the same to the goal
3	Slightly important	One evaluation element is slightly more important than another evaluation element
5	Obviously important	One evaluation element is obviously more important than another evaluation element
7	Strongly important	One evaluation element is strongly more important than another evaluation element
9	Extremely important	One evaluation element is extremely more important than another evaluation element
2, 4, 6, 8	Median of two adjacent degrees	Used when the compromise is needed

(2) Construct the comparison judgment matrix (Equations 7, 8).


(7) $$B={\left({\text{b}}_{\text{ij}}\right)}_{\text{n}\times \text{n}}=\left[\begin{array}{cc} \begin{array}{cc} \begin{array}{c} B_{11}\\ B_{21} \end{array} & \begin{array}{c} B_{12}\\ B_{22} \end{array} \end{array} & \begin{array}{cc} \begin{array}{c} \ldots \\ \ldots \end{array} & \begin{array}{c} B_{1n}\\ B_{21} \end{array} \end{array}\\ \begin{array}{cc} \begin{array}{c} \vdots \\ B_{n1} \end{array} & \begin{array}{c} \vdots \\ B_{n2} \end{array} \end{array} & \begin{array}{cc} \begin{array}{c} \vdots \\ \ldots \end{array} & \begin{array}{c} \vdots \\ B_{nn} \end{array} \end{array} \end{array}\right]$$

(8) $$b_{ij}>0,b_{ji}=\frac{1}{b_{ij}},b_{ii}=1$$

Where *b_ij_* represents the importance of criterion layer *B_i_* compared to criterion layer *B_j_* in relation to the objective layer, with values determined using the “1–9 Scale Method “ outlined in **Table 3**, n is the number of indicators in the criterion layer, and the results are shown in **Supplementary Tables S4**-**S8**.

**Table 3:** Effects of biochar on net income of potatoes in 2023 and 2024.

Year	Treatments		Water fee (CNY ha^-1^)	Fertilizer input (CNY ha^-1^)	Other input(CNY ha^-1^)	Gross income (CNY ha^-1^)	Net income (CNY ha^-1^)
	T	C					
2023	CK		580	4535	12000	61840	44725
	T1	C1	580	22535	12000	67696	32581
		C2	580	40535	12000	69808	16693
		C3	580	58535	12000	64480	-6635
	T2	C1	580	22535	12000	70928	35813
		C2	580	40535	12000	76640	23525
		C3	580	58535	12000	65840	-5275
	T3	C1	580	22535	12000	65680	30565
		C2	580	40535	12000	65536	12421
		C3	580	58535	12000	62752	-8363
2024	CK		580	4535	12000	56192	39077
	T1	C1	580	4535	12000	63056	45941
		C2	580	4535	12000	65728	48613
		C3	580	4535	12000	59616	42501
	T2	C1	580	4535	12000	67472	50357
		C2	580	4535	12000	73440	56325
		C3	580	4535	12000	61632	44517
	T3	C1	580	4535	12000	60800	43685
		C2	580	4535	12000	59920	42805
		C3	580	4535	12000	58640	41525
Source of variation				Significance levels of ANOVA			
T			–	–	–	**	*
C			–	–	–	**	ns
T × C			–	–	–	**	*

Mark the same as mentioned above. *means significant difference (P< 0.05) while ns means no significant difference (p > 0.05).

(3) Calculation of Indicator Weights

The normalized weight coefficient *M_i_* for the criterion layer indicator *B_i_* is calculated using the following formula (Equations 9, 10):


(9) $$M_{i}=\prod_{j=1}^{n}B_{i}/B_{j}$$

(10) $$W_{AHP}=\frac{\sqrt[{n}]{M_{i}}}{\sum_{i=1}^{n}\sqrt[{n}]{M_{i}}}$$

Where *M_i_* represents the product of the elements in each row of the judgment matrix, using the same method, the weights for the standard layer and the indicator layer are calculated to obtain the overall weights of the indicators.


(11) $$W_{S}=W_{I}\cdot W_{\text{II}}$$

Where *W_I_* represents the weight of the indicator relative to the standard layer, while *W_II_* denotes the weight of the secondary indicator relative to the objective layer, these two weights are derived from judgment matrices at different levels and are calculated using formulas (1-8), with results presented in **Supplementary Table S9** (Equation 11).

(4) Consistency Check

The judgment matrix involves pairwise comparisons of multiple indicators, which may lead to contradictory situations and deteriorate consistency. Therefore, a consistency index is introduced (Equations 12–15).


(12) $$CI=\frac{\lambda_{max}-M\;}{M-1}$$

Where λ_max_ represents the maximum eigenvalue of the judgment matrix, and M is the order of the matrix.


(13) $$\lambda_{max}=\sum_{i=1}^{n}\lambda_{i}/M$$

(14) $$\lambda_{i}=\frac{\sum_{i=1}^{n}B_{ij}M_{j}}{M_{i}}$$

(15) $$CR=\frac{CI\;}{RI}$$

Where λ is the characteristic root of the judgment matrix, M_i_ is the weight value, and RI is the average random consistency index, as shown in **Table 4**. If CR<0.1, the consistency check passes; otherwise, the judgment matrix must be adjusted.

**Table 4:** Average consistency random index value.

Matrix order	1	2	3	4	5	6	7	8	9	10
RI	0	0	0.58	0.9	1.12	1.24	1.32	1.41	1.45	1.49

After the consistency check, each judgment matrix’s consistency index (CI) and consistency ratio (CR) are both less than 0.10, indicating satisfactory consistency among the judgment matrices. The priority order of the indicators is logically coherent.

#### 2.5.2 Determination of sub-factor weights using the entropy method

The entropy method is a weighting approach based on the amount of information provided by each indicator, characterized by a high degree of objectivity, effectively mitigating the influence of subjective factors on the weights. This method can describe evaluation indicators’ information content and degree of dispersion. The greater the dispersion, the higher the subjective weight of the indicator. Data standardization is required to eliminate the impact of measurement units on the results (Equation 16).


(16) $$G_{ij}=\frac{x_{ij}-min\left\{x_{1j},x_{2j},.,x_{nj}\right\}\;}{max\left\{x_{1j},x_{2j},.,x_{nj}\right\}-min\left\{x_{1j},x_{2j},.,x_{nj}\right\}}$$

(1) Calculate the normalized value of the i evaluation object under the j evaluation indicator (Equation 17).


(17) $$P_{ij}=\frac{x_{ij}}{\sum_{i=1}^{m}x_{ij}}$$

(2) Calculate the entropy value of the j indicator (Equation 18).


(18) $$e_{j}=-\frac{1}{lnm}\sum_{i=1}^{m}P_{ij}lnP_{ij}$$

(3) Calculate the deviation degree of the j indicator (Equation 19).


(19) $$d_{j}=1-e_{j}$$

(4) Normalize the deviation degree of the j indicator, which is the weight given by (Equation 20):


(20) $$W_{EM}=\frac{d_{j}}{\sum_{i=1}^{m}d_{k}}$$

#### 2.5.3 Combination weighting method

A linear combination of objective weights and subjective weights can obtain the comprehensive weight (Equation 21):


(21) $$W_{AM}=\alpha_{1}\cdot W_{1}+\alpha_{2}\cdot W_{2}$$

where *α_1_* and *α_2_* are the combination coefficients, with *α_1_*, *α_2_* > 0 and *α_1_* + *α_2_* = 1. In this study, W1 and W2 represent the AHP weight (**Supplementary Table S9**) and EM weight (**Supplementary Table S10**) as provided by Zhu et al. (2021), with the results shown in **Supplementary Table S11**.

The goal of establishing the objective function for optimization is to minimize the deviation of the comprehensive weights. Based on the properties of matrix differentiation, the model can be transformed as follows (Equation 22):


(22) $$\left[\begin{array}{cc} W_{1}^{T}W_{1} & W_{1}^{T}W_{2}\\ W_{2}^{T}W_{1} & W_{2}^{T}W_{2} \end{array}\right]\left[\begin{array}{c} \alpha_{1}\\ \alpha_{2} \end{array}\right]=\left[\begin{array}{cc} W_{1}^{T} & W_{1}\\ W_{2}^{T} & W_{2} \end{array}\right]$$

#### 2.5.4 Fuzzy comprehensive evaluation method

This study established ten evaluation indicators and five comprehensive performance levels—”Excellent, Good, Average, Poor, and Very Poor”— n=10 and m=5, respectively. For positive indicators, a more significant value indicates a better performance, while for negative indicators, a smaller value is preferred (**Supplementary Table S12**).

The evaluation indicators are categorized into positive and negative indicators. The membership degrees are calculated using linear functions based on trapezoidal and triangular distributions. Then, a fuzzy comprehensive evaluation matrix R is constructed according to each membership degree.

Membership Function for Positive Indicators (Higher is Better):

Excellent level membership function (Equation 23):


(23) $${\text{M}}_{\text{Z}1}\left(x\right)=\{\begin{array}{c} 1,\;\;\;\;\;\;x\geq C_{1}\\ \frac{x-C_{2}}{C_{1}-C_{2}},\;\;\;\;\;\;C_{2}<x<C_{1}\\ 0,\;\;\;\;\;\;x\ll C_{2} \end{array}$$

Membership Functions for Good, Average, and Poor Levels (Equation 24):


(24) $${\text{M}}_{\text{Ze}}\left(x\right)=\{\begin{array}{c} 1,\;\;\;\;\;\;x=C_{e}\\ \frac{x-C_{e+1}}{C_{e}-C_{e+1}},\;\;\;\;\;\;C_{e+1}<x<C_{e}\\ \frac{C_{e-1}-x}{C_{e-1}-C_{e}},\;\;\;\;\;\;C_{e}<x<C_{e-1}\\ 0,\;\;\;\;\;\;x\ll C_{e+1},x\geq C_{e-1} \end{array}$$

Poor Level Membership Function (Equation 25):


(25) $${\text{M}}_{\text{Z}5}\left(x\right)=\{\begin{array}{c} 1,\;\;\;\;\;x\ll C_{5}\\ \frac{C_{4}-x}{C_{4}-C_{5}},\;\;\;\;\;\;C_{5}<x<C_{4}\\ 0,\;\;\;\;\;x\geq C_{4} \end{array}$$

For inverse indicators, where a smaller value indicates a better performance, the membership functions are defined as follows:

Excellent level membership function:


(26) $${\text{M}}_{\text{Z}1}\left(x\right)=\{\begin{array}{c} 1,\;\;\;\;\;\;x\ll C_{1}\\ \frac{C_{2}-x}{C_{2}-C_{1}},\;\;\;\;\;\;C_{1}<x<C_{2}\\ 0,\;\;\;\;\;\;x\geq C_{2} \end{array}$$

Membership Functions for Good, Average, and Poor Levels:


(27) $${\text{M}}_{\text{Ze}}\left(x\right)=\{\begin{array}{c} 1,\;\;\;\;\;\;x=C_{e}\\ \frac{C_{e+1}-x}{C_{e+1}-C_{e}},\;\;\;\;\;\;C_{e}<x<C_{e+1}\\ \frac{x-C_{e-1}}{C_{e}-C_{e-1}},\;\;\;\;\;\;C_{e-1}<x<C_{e}\\ 0,\;\;\;\;\;\;x\ll C_{e-1},x\geq C_{e+1} \end{array}$$

Poor Level Membership Function:


(28) $${\text{M}}_{\text{Z}5}\left(x\right)=\{\begin{array}{c} 1,\;\;\;\;\;\;x\geq C_{5}\\ \frac{x-C_{4}}{C_{5}-C_{4}},\;\;\;\;\;\;C_{4}<x<C_{5}\\ 0,\;\;\;\;\;\;x\ll C_{4} \end{array}$$

Based on the membership function formulas for each evaluation indicator, we construct the fuzzy comprehensive evaluation matrix R that reflects the degree of membership for each indicator to the evaluation levels.

The “weighted average-type” operator combines the weight matrix A obtained from the Analytic Hierarchy Process (AHP) and the Entropy Method with the fuzzy comprehensive evaluation matrix R to obtain the fuzzy comprehensive evaluation result vector B, expressed as follows (Equations 28, 29):


(28) $$B=A*R=\left\{b_{1},b_{2},\ldots ,b_{m}\right\}$$

(29) $$b_{j}=min\left\{1,\sum_{i=1}^{n}a_{i}r_{ij}\right\}\;\;\;\;j=1,2,3\ldots ,m$$

The maximum membership degree method is employed to rate the results of the fuzzy comprehensive evaluation (Equation 30):


(30) $$b_{k}=max\left\{b_{1},b_{2},\ldots ,b_{m}\right\}$$

#### 2.5.5 Calculation of multi-level fuzzy evaluation values

The calculation formula for the fuzzy comprehensive evaluation result vector B is as follows (Equation 31):


(31) $$B=W_{AM}\cdot R=\;\left\{b_{1},b_{2},\ldots ,b_{5}\right\}$$

Where *R* is the fuzzy comprehensive evaluation matrix, and *b_1_,b_2_*,…, and *b_5_* represent the membership degrees of different evaluation levels.

To further analyze the results, the semantic scale (Excellent, Good, Average, Poor, Very Poor) is quantified and assigned values of 5, 4, 3, 2, and 1, respectively. These values are multiplied by B to obtain the comprehensive scores for each treatment.

### 2.6 Data processing and analysis

Data was statistically organized using Excel 2016, and calculations for the analytic hierarchy process (AHP) and entropy method were performed. SPSS 20.0 was utilized for multiple comparisons (Multiple Comparisons: Least Significant Difference), while Origin 2021 was used for plotting.

## 3 Results and analysis

### 3.1 Effects of biochar application on potato growth, yield and net income

The results of the two-way ANOVA indicated that the pyrolysis temperature and application rate of biochar, as well as their interaction, significantly affected various growth indices, yield, and net income of potatoes (*P*< 0.05; **Tables 5**, **3**). During the 2023–2024 period, the dry matter weight, plant height, stem diameter, leaf area index (LAI), SPAD value, and yield of potatoes generally showed a trend of first increasing and then decreasing with increasing pyrolysis temperature and application rate of biochar. Among the treatments, the biochar produced at the medium pyrolysis temperature (500°C) applied at a rate of 20 t ha^-1^ performed best, resulting in a yield increase of 8.44% to 27.15% compared to other treatments (*P*< 0.05; average values for 2023 and 2024). In 2023, the net income from potatoes was highest under the treatment without biochar application (CK), whereas in 2024, applying the same temperature biochar (500°C) at a rate of 20 t ha^-1^ yield the highest net income. Overall, the average net income of potatoes in 2023 and 2024 under the C2T2 treatment was the most favorable, showing an increase of 2.83% to 159.85% compared to other treatments.

**Table 5:** Effects of biochar on aboveground dry matter, tuber dry matter, root dry matter, plant height, stem diameter, leaf srea index (LAI), SPAD value, and yield in 2023 and 2024.

Year	Treatments		Shoot dry matter (t ha^-1^)	Tuber dry matter (t ha^-1^)	Root dry matter (t ha^-1^)	Plant height (cm)	Stem diameter (mm)	LAI (m^2^ m^-2^)	SPAD	Yield (t ha^-1^)
	T	C								
2023	CK		5.17e	10.12f	0.138d	75.65d	16.10e	3.12e	43.50d	38.65d
	T1	C1	5.85cd	11.82c	0.153abc	85.53c	19.14c	3.56cd	47.98c	42.31bc
		C2	6.62b	13.26b	0.155ab	94.67b	19.95bc	3.95b	53.90b	43.63bc
		C3	5.53de	10.87de	0.145cd	80.60cd	17.10de	3.34cd	46.00cd	40.30cd
	T2	C1	6.76ab	13.60ab	0.157ab	99.92ab	20.65ab	4.05ab	58.90a	44.33b
		C2	7.22a	14.38a	0.160a	105.21a	22.67a	4.28a	62.30a	47.90a
		C3	5.76cd	11.35d	0.151ab	83.21c	17.63d	3.52c	46.95c	41.15cd
	T3	C1	5.56de	11.00de	0.148bc	80.95cd	17.13de	3.38cd	46.70c	41.05cd
		C2	5.48de	10.51ef	0.144cd	78.73cd	16.89de	3.30de	45.53cd	40.96cd
		C3	5.30e	10.21ef	0.143cd	76.98d	16.53de	3.25de	44.93cd	39.22d
2024	CK		4.56e	9.27e	0.134d	71.98f	15.72e	2.99e	40.23e	35.12f
	T1	C1	5.22cd	11.06c	0.152abc	83.27c	18.88c	3.47c	45.43c	39.41cd
		C2	5.93b	12.51b	0.154ab	90.91b	19.67bc	3.83b	51.78b	41.08bc
		C3	4.94d	9.93de	0.144bc	77.09def	16.91d	3.22d	43.26cd	37.26ef
	T2	C1	6.02ab	12.42ab	0.156ab	97.26ab	20.53ab	3.91ab	55.57ab	42.17b
		C2	6.51a	13.74a	0.158a	102.77a	22.39a	4.18a	58.35a	45.90a
		C3	5.23cd	10.62cd	0.149abc	80.63cd	17.31cd	3.46c	44.24cd	38.52cde
	T3	C1	4.97d	10.15d	0.146bc	77.45e	17.00cd	3.30cd	43.85cd	38.00de
		C2	4.85de	9.87de	0.143cd	75.68def	16.54de	3.17de	42.69cde	37.45ef
		C3	4.75de	9.75de	0.142cd	74.34ef	16.36de	3.14de	41.85de	36.65ef
Source of variation			Significance levels of ANOVA							
T			**	**	**	**	**	**	**	**
C			**	**	**	**	**	**	*	**
T × C			**	**	**	**	**	**	**	**

Different lowercase letters indicate significant differences among all the treatments at the 0.05 level. ** means an extremely significant difference (P< 0.01).

### 3.2 Effects of biochar application on potato quality, accumulation of NPK in different organs, and water-nutrient use efficiency

**Figure 3:**
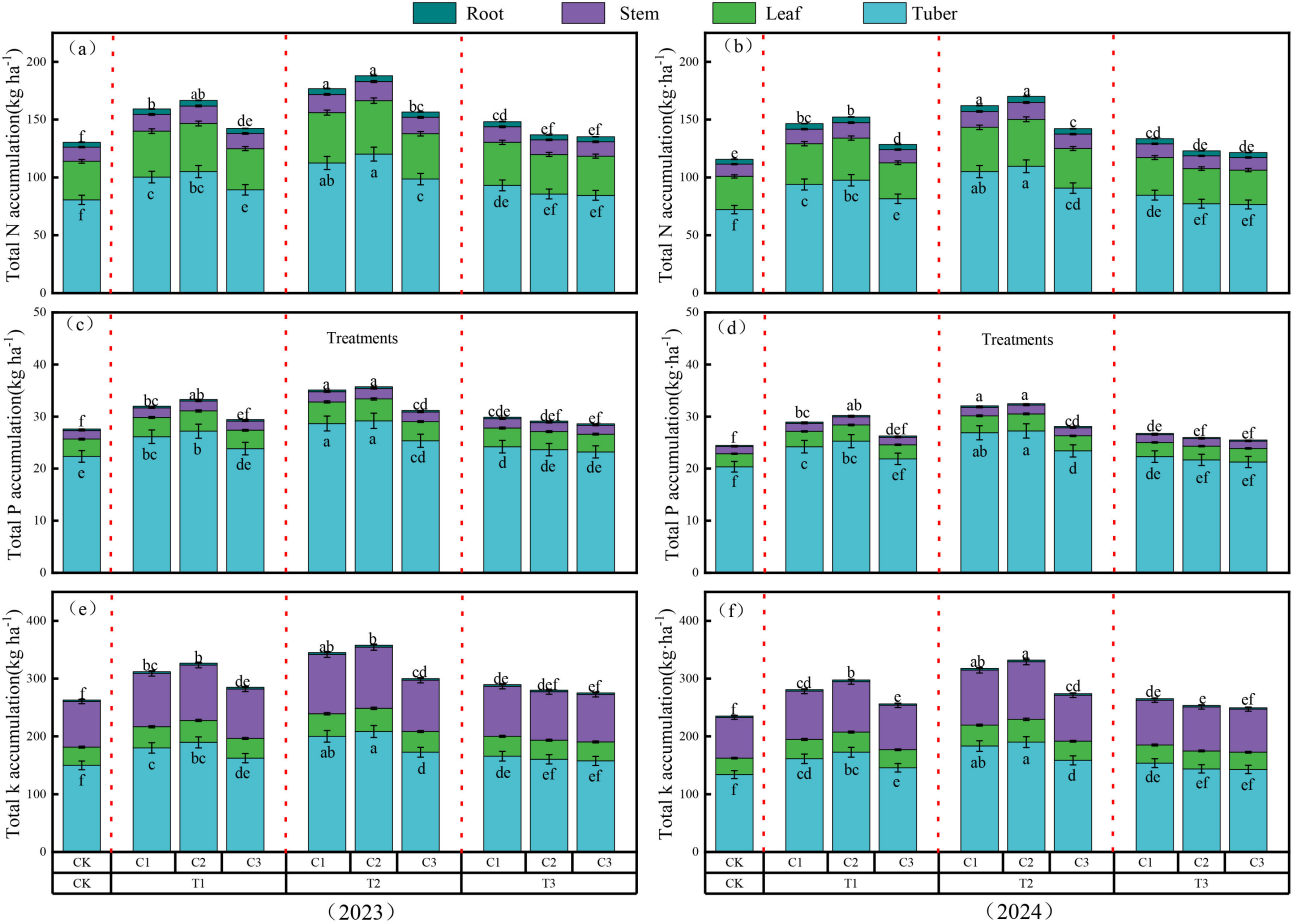
Bar charts showing the total accumulation of N, P, and K in different plant organs under varying pyrolysis temperatures and application rates in 2023 and 2024. Different letters above the bars indicate significant differences in the total N **(a, b)**, P **(c, d)**, and K **(e, f)** accumulation in the whole plant, while different letters below the bars indicate significant differences in N, P, and K accumulation in the tubers (P < 0.05).

The results of the two-way ANOVA indicated that the pyrolysis temperature and application rate of biochar, as well as their interaction, had significant effects on potato quality, nutrient accumulation in different organs, and water-nutrient use efficiency (*P*< 0.05; **Tables 6**, **7**). During the 2023–2024 period, the starch content, vitamin C content, and nitrogen, phosphorus, and potassium accumulation in various organs of potatoes generally showed a trend of increasing and then decreasing with increasing biochar pyrolysis temperature and application rate. Among the treatments, biochar produced at the medium pyrolysis temperature (500°C) at an application rate of 20 t ha^-1^ performed best, with the C2T2 treatment showing starch content and vitamin C content higher by 2.50% to 19.38% and 1.97% to 23.30%, respectively (**Table 6**, average values for 2023 and 2024). The total accumulation of nitrogen, phosphorus, and potassium in the C2T2 treatment was higher by 5.67% to 45.49%, 1.57% to 30.96%, and 4.12% to 38.46%, respectively (**Figure 3**, average values for 2023 and 2024). Additionally, the water-nutrient use efficiency in the C2T2 treatment was significantly higher than in other therapies by 10.03% to 30.20% and 8.44% to 27.15% (**Table 7**, *P*< 0.05; average values for 2023 and 2024).

**Table 6:** Effects of biochar on potato quality in 2023 and 2024.

Year	Treatments		Starch content (%)	Reducing sugar content (%)	Vitamin C content (mg/(100 g FW))
	T	C			
2023	CK		16.61d	0.430a	19.28d
	T1	C1	18.40b	0.353d	21.89bc
		C2	19.08ab	0.325e	22.55ab
		C3	17.46cd	0.397bc	20.33cd
	T2	C1	19.12ab	0.317ef	23.06ab
		C2	19.65a	0.300f	23.64a
		C3	17.90bc	0.370cd	21.29c
	T3	C1	17.71cd	0.385c	20.62cd
		C2	17.03cd	0.400bc	20.07cd
		C3	16.83cd	0.420ab	19.61d
2024	CK		16.08e	0.424a	18.53e
	T1	C1	18.32abc	0.337d	21.62b
		C2	18.70ab	0.310e	22.05ab
		C3	17.27d	0.378c	19.75cde
	T2	C1	18.95ab	0.302ef	22.66a
		C2	19.37a	0.286f	22.98a
		C3	17.54cd	0.352cd	20.88bc
	T3	C1	17.71bcd	0.363bc	20.13cd
		C2	16.85de	0.387abc	19.61cde
		C3	16.47de	0.408a	19.25de
Source of variation				Significance levels of ANOVA	
T			**	**	**
C			**	**	**
T× C			**	**	**

Mark the same as mentioned above.

**Table 7:** Effects of biochar on water-nutrient use efficiency in potatoes in 2023 and 2024.

Years	Treatments		WUE (kg m^3^)	PFP (kg kg^-1^)
	T	C		
2023	CK		11.98d	49.55d
	T1	C1	13.14bc	54.24bc
		C2	13.67b	55.94bc
		C3	12.52cd	51.67cd
	T2	C1	13.84b	56.83b
		C2	15.11a	61.41a
		C3	12.76cd	52.76cd
	T3	C1	12.74cd	52.63cd
		C2	12.72cd	52.51cd
		C3	12.19d	50.28d
2024	CK		11.26f	45.03f
	T1	C1	12.74cd	50.53cd
		C2	13.43bc	52.67bc
		C3	12.34de	47.77ef
	T2	C1	13.65b	54.06b
		C2	15.14a	58.85a
		C3	12.44de	49.38cde
	T3	C1	12.26de	48.72de
		C2	12.13de	48.01ef
		C3	11.82ef	46.99ef
Source of variation			Significance levels of ANOVA	
T			**	**
C			**	**
T × C			**	**

Mark the same as mentioned above.

In contrast, the reduced sugar content of the potatoes showed a declining trend. Under the medium pyrolysis temperature (500°C) and an application rate of 20 t ha^-1^, The reducing sugar content of the C2T2 treatment decreased by 5.33% to 31.38% compared to other treatments. (**Table 6**, average values for 2023 and 2024).

### 3.3 Effects of biochar application on soil nitrate nitrogen, available phosphorus, and available potassium accumulation

**Figure 4:**
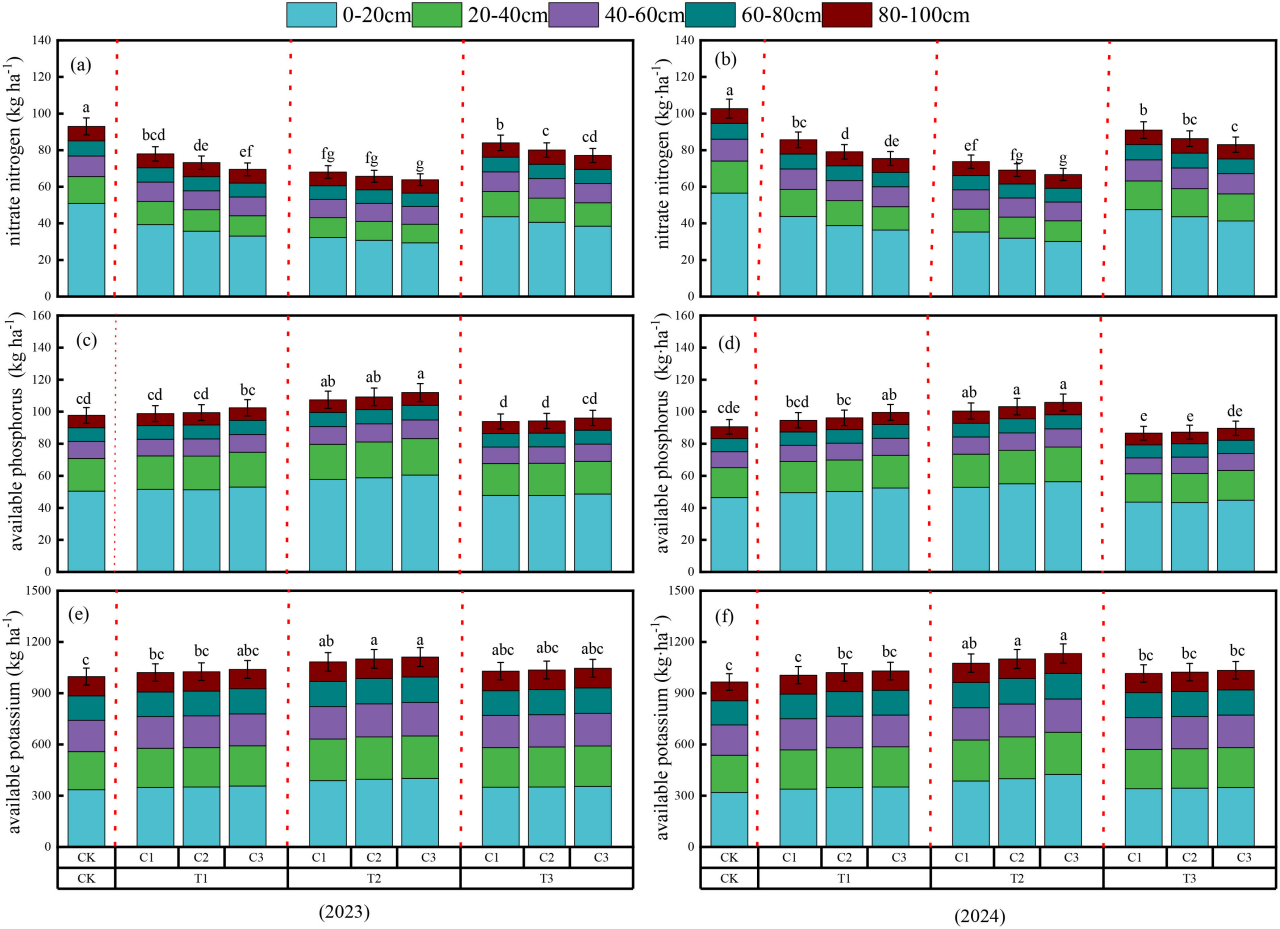
Accumulation of nitrate nitrogen **(a, b)**, available phosphorus **(c, d)**, and available potassium **(e, f)** in different soil layers (0–20 cm, 20–40 cm, 40–60 cm, 60–80 cm, and 80–100 cm) at harvest under various pyrolysis temperatures and application rates in 2023 and 2024. Different lowercase letters above the bars indicate significant differences in total accumulation (0–100 cm soil depth) among treatments according to Duncan’s multiple range test (P < 0.05).

The results of the two-way ANOVA indicated that the pyrolysis temperature and application rate of biochar, as well as their interaction, significantly affected the accumulation of nitrogen, phosphorus, and potassium in the soil (*P*< 0.05; **Figure 4**). Between 2023 and 2024, the nitrate nitrogen content in the 0~100 cm soil profile showed a trend of first decreasing and then increasing with increasing biochar pyrolysis temperature, while it gradually decreased with increasing application rate. At a medium pyrolysis temperature (500°C), the nitrate nitrogen content at an application rate of 30 t ha^-1^ was relatively low, being higher by 3.28% to 49.89% compared to the C3T2 treatment (average values for 2023 and 2024).

The accumulation of available phosphorus and potassium in the soil showed a trend of increasing and then decreasing with increasing biochar pyrolysis temperature. In contrast, it gradually increased with increasing application rate. At a high pyrolysis temperature (700°C), the accumulation of available phosphorus at an application rate of 10 t ha^-1^ was low, while the accumulation of available potassium was lowest under control conditions. The accumulation of available phosphorus in all treatments was higher by 0.55% to 20.65% compared to the C1T3 treatment (average values for 2023 and 2024), and the accumulation of available potassium was significantly higher than the control treatment by 3.83% to 13.24% (average values for 2023 and 2024). Additionally, soil nitrate nitrogen, available phosphorus, and potassium accumulation were primarily concentrated in the 0~40 cm soil layer. This may be related to the duration of biochar application and soil incorporation depth.

### 3.4 Effects of the interaction between biochar pyrolysis temperature and application rate on the potato PFCE model

To comprehensively evaluate the feasibility of the ten treatments, a multi-level fuzzy comprehensive evaluation (PFCE) model was employed. Given that yield and economic returns are the primary concerns of farmers, tuber quality is essential for processing and consumption, soil nutrient residues reflect agricultural environmental impacts, and water and fertilizer use efficiency are critical for sustainable production in arid regions, ten evaluation indicators were selected: yield (Z_11_), net income (Z_12_), starch content (Z_21_), vitamin C content (Z_22_), reducing sugar content (Z_23_), water use efficiency (WUE, Z_31_), partial factor productivity of fertilizer (PFP, Z_32_), soil nitrate nitrogen accumulation (Z_41_), soil available phosphorus accumulation (Z_42_), and soil available potassium accumulation (Z_43_).

Subjective and objective weights were calculated using the AHP and Entropy Method (EM) (**Supplementary Tables S9**, **S10**). The reliability of WAHP was tested using Equation 15. Due to the data-driven nature of the entropy method, there were differences in WEM between the two years. Finally, WAHP and WEM were combined to form composite weights using Equation 21 and Equation 22 (**Supplementary Table S11**).

The multi-level fuzzy evaluation values are presented in **Supplementary Table S13**. In 2023, at the same level of biochar application, the composite evaluation value gradually increased when the pyrolysis temperature was below 500°C. Conversely, under the same pyrolysis temperature conditions, the evaluation value continuously decreased with increasing application rate In 2023, CK was more favorable for improving the comprehensive evaluation. In 2024, the comprehensive benefits of potatoes showed a trend of increasing and then decreasing with rising pyrolysis temperature and application rate. The maximum comprehensive benefit was achieved when the biochar pyrolysis temperature was 500°C, and the application rate was 20 t ha^-1^.

**Figure 5:**
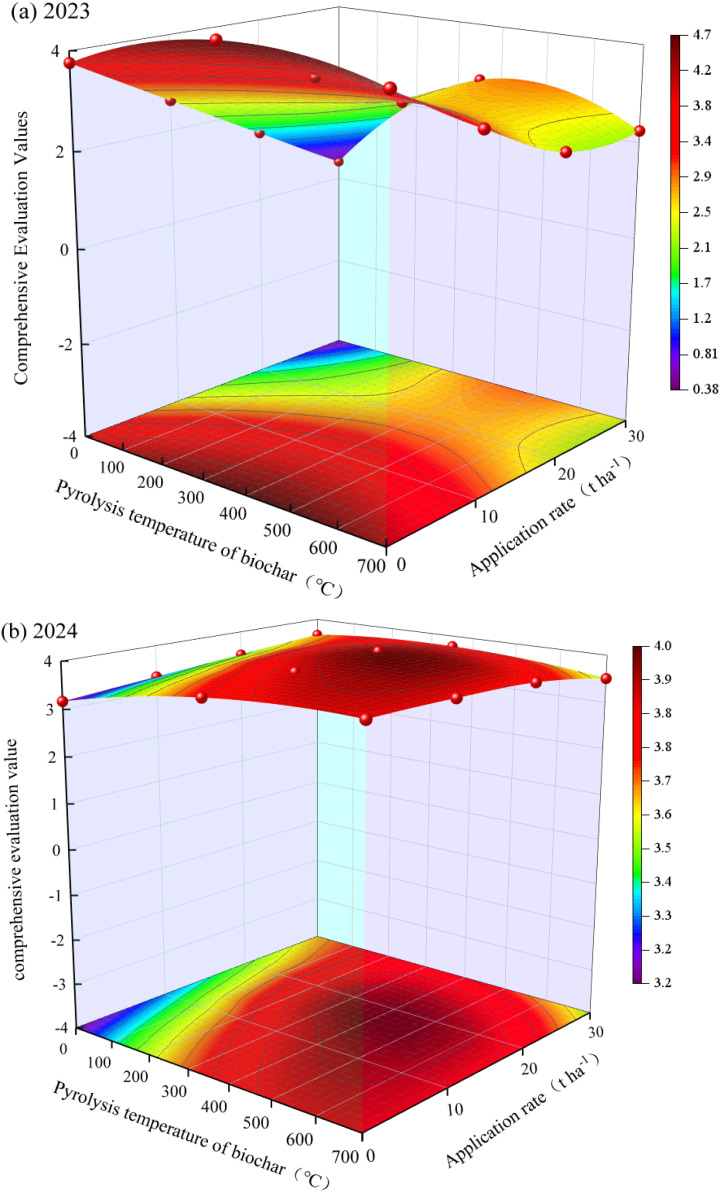
The Interactive Effects of Biochar Pyrolysis Temperature and Application Rate on the Comprehensive Evaluation Values of Potatoes in 2023 **(a)** and 2024 **(b)**.

Based on the multi-level fuzzy evaluation values, the quadratic surface coefficients of determination (*R*²) established with biochar pyrolysis temperature and application rate were 0.82 and 0.80 for 2023 and 2024, respectively (**Figure 5**), indicating that the regression model effectively explains the impact of biochar pyrolysis temperature and application rate on the overall benefits of potatoes. Based on the comprehensive model from multi-level fuzzy evaluation and combined with actual production management, it was concluded that the optimal multi-objective comprehensive level is achieved when the biochar pyrolysis temperature is 400–500°C, and the application rate is 18–20 t ha^-1^.

## 4 Discussion

### 4.1 Effects of biochar on potato growth, yield, and net income

Multiple studies have shown that the application of biochar can promote crop growth, increase dry matter quality, enhance the accumulation of nitrogen, phosphorus, and potassium in plants, and improve crop yield and quality (Alkharabsheh et al., 2021; Iqbal et al., 2019; Kapoor et al., 2022; Khan et al., 2021a, b). A recent meta-analysis pointed out that biochar produced at relatively low pyrolysis temperatures (≤400°C and 401-500°C) has a more favorable effect on crop productivity (Rehman et al., 2021). This study indicates that when the application rate of biochar is constant, potato dry matter, plant height, stem diameter, leaf area index (LAI), SPAD values, accumulation of nitrogen, phosphorus, and potassium in the plant, and tuber yield all initially increase and then decrease with rising biochar pyrolysis temperatures (**Figure 3**, **Table 5**). This trend may be attributed to biochar’s relatively stable aromatic structure produced at moderate pyrolysis temperatures, which contains more C=O and C-H functional groups that can serve as nutrient exchange sites (Tomczyk et al., 2020). This facilitates better retention of nutrients in the soil, thereby enhancing the crop’s absorption of nitrogen, phosphorus, and potassium from fertilizers (Tanure et al., 2019). Additionally, biochar produced at moderate pyrolysis temperatures has a higher content of essential nutrient elements (Na, K, Ca, and Mg) on its surface, which increases soil nutrient levels (Xi et al., 2020). In contrast, biochar produced at high pyrolysis temperatures releases excessive soluble nutrients, resulting in a decline in plant photosynthetic capacity and reduced crop yields (Gao et al., 2021). Moreover, biochar produced at high pyrolysis temperatures has a higher C/N ratio, which can reduce soil enzyme activity (Khadem and Raiesi, 2017) and negatively impact the soil microbial environment, inhibiting crop growth and ultimately lowering yields (Brtnicky et al., 2021). This study also found that when the pyrolysis temperature of biochar is constant, potato tuber yield increases with biochar application rates from 0 to 20 t ha^-1^, but further increases in application rates do not lead to higher yields and may even cause a decline (**Table 5**). This could be due to excessive biochar application leading to decreased microbial activity, thereby inhibiting the crop’s nutrient uptake (Xiang et al., 2021; Semida et al., 2019). Additionally, the economic cost associated with large-scale biochar application may limit its commercialization and use in agriculture (Farhangi-Abriz et al., 2021).

Two-way ANOVA indicated that the interaction between biochar pyrolysis temperature and application rate significantly affected potato dry matter, plant height, stem diameter, leaf area index (LAI), SPAD values, and yield (*P*<0.05) (**Table 5**). Therefore, selecting appropriate biochar pyrolysis temperatures and application rates is essential for maximizing potato yield. Overall, when the biochar pyrolysis temperature is set at 500°C and the application rate at 20 t ha^-1^, there is a significant increase in LAI, accumulation of dry matter in the stems and tubers, and promotion of root and canopy development, which enhances the root system’s ability to absorb water and nutrients, facilitates leaf formation, and ultimately results in significant yield benefits (Yang et al., 2022).

### 4.2 Effects of biochar on potato quality and water-fertilizer use efficiency

Biochar contains essential nutrients such as nitrogen, phosphorus, and potassium necessary for the physiological growth of potatoes. It can directly release soluble nutrients into the soil solution for crop uptake, thus influencing potato quality (Liu et al., 2018; Mokrani et al., 2018). Research by Jiao et al. (2018) indicates that adding biochar can absorb toxic antibiotics harmful to potato roots, increasing potatoes’ starch, fat, protein, and vitamin content, thereby enhancing tuber quality. Agegnehu et al. (2017) found that an appropriate amount of biochar can improve crop quality, while excessive application can lead to a decline in quality. Our study reached similar conclusions (**Table 6**), The excessive application of biochar may inhibit starch accumulation and reduce potato taste due to soil moisture excess or nutrient imbalance (Mahanjana, 2018). Suthar et al. (2018) found that fruits produced under low-temperature biochar (300°C) treatments were firmer, darker, and had higher sugar, acid, and vitamin C (ascorbic acid) content, leading to more excellent nutritional value. In contrast, our study indicates that biochar at a medium pyrolysis temperature (500°C) yields higher starch and vitamin content in potatoes. In comparison, reducing sugars are higher with low-temperature biochar treatment (300°C) (**Table 6**). These differences may be attributed to variations in soil texture, climatic conditions, and crop species.

This study indicates that the pyrolysis temperature and application rate of biochar and their interaction significantly affect water and nutrient use efficiency (**Table 7**). Overall, potato water and nutrient use efficiency show a trend of increasing and decreasing with the rise in biochar pyrolysis temperature and application rate, with C2T2 being optimal. This is consistent with the findings of Liu et al. (2022). As pyrolysis temperature increases, biochar’s surface area and porosity also increase, enhancing the soil’s ability to retain water and nutrients, thereby improving crop water and nutrient use efficiency (Tanure et al., 2019; Murtaza et al., 2021). However, excessively high pyrolysis temperatures can lead to increased hydrophobicity of biochar (Hassan et al., 2020), reducing its water retention capacity and inhibiting crop water uptake.

Additionally, high pyrolysis temperatures may cause excessive release of soluble nutrients, which can suppress photosynthetic capacity and decrease nutrient use efficiency (Gao et al., 2021). The application of biochar can significantly increase organic carbon content (Dong et al., 2016), demonstrating a compensatory effect in nutrient-deficient soils (Zhang et al., 2024), thereby enhancing nutrient efficiency and biomass in plants (Han et al., 2023). Furthermore, application rates exceeding 20 t ha^-1^ may inhibit soil microbial activity, negatively impacting dry matter accumulation and water and nutrient uptake and increasing input costs (**Table 3**). In summary, maintaining a moderate pyrolysis temperature (500°C) and an appropriate application rate (20 t ha^-1^) can maximize potato water and nutrient use efficiency and crop quality.

### 4.3 Effects of different pyrolysis temperatures and application rates on soil nitrate nitrogen, available phosphorus, and available potassium accumulation

Drought stress can inhibit potato plants’ nutrient absorption, leading to excessive nutrient residues in the soil (Rea et al., 2022). Research has shown that biochar can promote effective nutrient absorption while reducing nutrient residues and soil nutrient loss, decreasing the risk of nutrient leaching and groundwater contamination (El-Naggar et al., 2019). Our study found that as the pyrolysis temperature of biochar increases, soil NO_3_-N content initially decreases and then increases (**Figure 4**). This may be due to the larger specific surface area of biochar produced at higher temperatures, which provides more adsorption sites and spaces for nutrients, allowing for more effective adsorption of soil NO_3_-N (Zhang et al., 2021). However, excessively high pyrolysis temperatures significantly enhance the hydrophobicity of biochar surfaces, weakening their contact with NO_3_-N through hydrogen bonding (Sochacki et al., 2024).

Furthermore, applying high-temperature biochar may promote the decomposition of organic matter in the soil, releasing more ammonium nitrogen. As the supply of ammonium nitrogen increases, nitrifying bacteria convert more ammonium nitrogen into nitrate nitrogen, increasing soil nitrate nitrogen content (Dong et al., 2016). Under the same pyrolysis temperature conditions, the accumulation of soil nitrate nitrogen gradually decreases with increasing biochar application rates, primarily due to the strong adsorption capacity of the biochar itself (Mehmood et al., 2022). Additionally, the porous structure and larger specific surface area of biochar slow down the release of nutrient fertilizers (Das and Ghosh, 2023).

Our study found that the adequate phosphorus in the soil increased and then decreased with rising biochar pyrolysis temperatures, which is consistent with the findings of Khadem et al. (2021) (**Figure 4**). This may be because, as pyrolysis temperature increases, the volatile components of biochar decrease, pore size decreases, and porosity and specific surface area increase (Saghir et al., 2022). The porous structure of biochar provides a suitable habitat for microorganisms such as phosphate-solubilizing bacteria, which can help dissolve insoluble phosphates in the soil (Lu et al., 2023). However, despite the increased input of phosphorus from high-temperature biochar (700°C), the adequate phosphorus in the soil decreased. This may be due to the soil’s inability to effectively absorb the phosphorus added from biochar or the reaction of sufficient calcium in the soil with phosphorus, leading to its fixation and reduced availability (Lu et al., 2023). The trend in soil-effective potassium changes similarly to that of adequate phosphorus. This may be because, at lower pyrolysis temperatures, biochar retains more soluble potassium. When the pyrolysis temperature is moderate (500°C), potassium is more easily released from biochar into the soil, resulting in increased effective potassium content. However, as the pyrolysis temperature rises further, the mineral structure of biochar may change, particularly regarding potassium-related compounds, which may undergo chemical transformations to form less soluble or less available mineral forms, leading to a decrease in effective potassium content in the soil (Bilias et al., 2023). Gao et al. (2018) demonstrated that the soil’s adequate phosphorus and potassium levels increased with higher biochar application rates. Our study also found similar results (**Figure 4**). This is partly due to the inherent phosphorus and potassium content in biochar, which can be released into the soil; on the other hand, biochar’s high adsorption capacity not only helps retain phosphorus and potassium in the soil but also reduces the risk of these nutrients being lost due to leaching during rainfall or irrigation.

### 4.4 Multi-objective evaluation optimization

Many studies rely solely on subjective or objective single techniques to evaluate a particular indicator, lacking a comprehensive assessment of multiple indicators. Therefore, to overcome the shortcomings of previous research that only considers a single weight or a simple combination of subjective and objective weights, this study coordinated the impact of various indicators on the comprehensive benefits of potatoes. Firstly, this study employed the Analytic Hierarchy Process (AHP) and the Entropy Method to determine the factors and weights for economic, quality, water and fertilizer utilization efficiency, and environmental benefits of potatoes (S9 and S10). Subsequently, using principles of fuzzy mathematics, a multi-level fuzzy comprehensive evaluation was performed on the weights (**Supplementary Table S13**), concluding that the CK treatment in 2023 and the C2T2 treatment in 2024 were more beneficial for enhancing comprehensive benefits. In determining the weight indicators, this study considered the actual interests of local farmers, which resulted in a higher weight assigned to economic benefit indicators. Additionally, since biochar was only applied in the first year of the experiment, a high application rate significantly reduced the overall benefit value in the first year due to high costs. However, from a sustainable development perspective, the optimal treatment of C2T2 obtained through multi-level fuzzy comprehensive evaluation holds practical significance for actual production.

Moreover, existing research typically reflects the optimal treatment effects obtained under specific experimental conditions (Wang et al., 2021; Keabetswe et al., 2019). However, due to time and cost constraints, field experiments often involve a limited number of treatments, leading to insufficient accuracy and detail in the comprehensive evaluation results, which restricts a thorough understanding of treatment effects. To address this issue, we established a binary quadratic surface regression model for biochar pyrolysis temperature and potato application rates based on the evaluation results (**Figure 5**). Through model simulations, we found that combining pyrolysis temperatures between 400–500°C and application rates of 18–20 t ha^-1^ achieves the best multi-objective comprehensive outcomes.

## 5 Conclusions

1. Appropriate biochar pyrolysis temperature and application rate significantly promoted potato growth, enhanced the uptake and accumulation of nitrogen, phosphorus, and potassium, improved water use efficiency (WUE) and partial factor productivity (PFP), increased starch and vitamin C contents, and reduced the content of reducing sugars, thereby achieving a coordinated improvement in both potato yield and tuber quality.
2. Soil nutrient accumulation patterns varied across treatments: nitrate nitrogen accumulation was lowest under C3T2, available phosphorus under C1T3, and available potassium under CK. Notably, the optimal values for different indicators were not achieved under the same biochar pyrolysis temperature and application rate.
3. Multi-objective optimization using the Analytic Hierarchy Process (AHP), entropy weight method, and fuzzy comprehensive evaluation indicated that the C2T2 treatment achieved the best balance among high yield, quality, efficiency, and environmental benefits. A fitted model based on the results of the multi-level fuzzy comprehensive evaluation, combined with practical management considerations, showed that the optimal biochar pyrolysis temperature and application rate were 400–500 °C and 18–20 t·ha^-1^, respectively.

These findings provide a scientific basis and practical guidance for the rational application of biochar in potato farmland in the arid and semi-arid regions of northern China.

## Data Availability

The original contributions presented in the study are included in the article/**Supplementary Material**. Further inquiries can be directed to the corresponding authors.
