# Supplementary material for: A multi-level fuzzy comprehensive evaluation model to optimize biochar application schemes for potato cultivation in North China
Source: Front Plant Sci. 2025 Jun 5;16:1571305. doi: 10.3389/fpls.2025.1571305 (PMC12178125; doi:10.3389/fpls.2025.1571305)
Supplement: Supplementary file 1 [file DataSheet1.docx]

**Supporting Information**

Number of Tables: 13

Number of figures: 1

**Fig. S1.** Daily air temperature (a), precipitation (b), wind speed (c), and humidity (d) from 2023–2024.

**Table S1.** Physicochemical Characteristics of Biochar under Different Pyrolysis Temperature Conditions

| Biochar property | Biochar species | | |
| --- | --- | --- | --- |
|  | T1 | T2 | T3 |
| Ash（%） | 9.27±0.4c | 13.53±0.8b | 15.22±0.13a |
| productivity（%） | 45.33±1.2a | 30.18±1.0b | 28.15±0.9c |
| EC | 1.82± 0.03c | 2.35 ± 0.18b | 3.56 ± 0.29a |
| pH | 7.2 ± 0.1c | 9.0± 0.1b | 10.2 ± 0.1a |
| C（%） | 58.23±0.3c | 60.12±0.2b | 65.18±0.5a |
| H（%） | 3.95±0.03a | 3.43±0.09b | 2.33±0.08c |
| N（%） | 2.05±0.08a | 1.83±0.03b | 1.55±0.03c |
| O（%） | 20.38±1.02a | 16.55±0.76b | 10.16±0.08c |
| H/C | 0.07±0.01a | 0.06±0.01b | 0.04±0.02c |
| O/C | 0.35±0.01a | 0.28±0.03b | 0.16±0.03c |
| TOC (g kg^−1^) | 401.22±0.9c | 450.08±1.1b | 502.38±1.8a |
| Total P (g kg^−1^) | 1.87±0.01c | 2.65±0.03b | 3.82±0.07a |
| Total K (g kg^−1^) | 37.22±0.12b | 43.56±0.18b | 50.29±0.20a |

**Note:** T1 = Biochar produced at 300 °C; T2 = Biochar produced at 500 °C; T3 = Biochar produced at 700 °C.Different lowercase letters indicate significant differences among all the treatments at the 0.05 level.

**Table S2.** Experimental design

| treatment | Pyrolysis temperature of biochar (℃) | Amount of biochar applied (t ha^-1^) |
| --- | --- | --- |
| CK | — | — |
| C1T1 | 300 | 10 |
| C2T1 | 300 | 20 |
| C3T1 | 300 | 30 |
| C1T2 | 500 | 10 |
| C2T2 | 500 | 20 |
| C3T2 | 500 | 30 |
| C1T3 | 700 | 10 |
| C2T3 | 700 | 20 |
| C3T3 | 700 | 30 |

**Note:** CK = Control; T1 = Biochar produced at 300 °C; T2 = Biochar produced at 500 °C; T3 = Biochar produced at 700 °C; C1 = 10 t ha^-1^ biochar application; C2=20 t ha^-1^ biochar application; C3 = 30 t ha^-1^ biochar application

**Table S3.** Irrigation Amounts for Each Growth Stage of Potato

| Growth stage of potato | 2023 | Irrigation quantity（mm） | 2024 | Irrigation quantity（mm） |
| --- | --- | --- | --- | --- |
| Seedling stage | 6/17 | 11.25 | 6/19 | 11.25 |
| Seedling stage—tuber initiation stage | 6/26 | 11.25 | 7/4 | 11.25 |
| tuber initiation stage | 7/6 | 21 | 7/13 | 21 |
| tuber initiation stage | 7/17 | 21 | 7/20 | 21 |
| tuber initiation stage-tuber bulking stage | 7/25 | 31.5 | 7/27 | 31.5 |
| tuber bulking stage | 8/5 | 31.5 | 8/6 | 31.5 |
| tuber bulking stage | 8/13 | 31.5 |  |  |
| tuber bulking stage—accumulation stage | 8/22 | 21 |  |  |

**Table S4.** Judgment Matrix and Criterion Layer Weights

| Criterion | Z_1_ | Z_2_ | Z_3_ | Z_4_ | Weights |
| --- | --- | --- | --- | --- | --- |
| Z_1_ | 1 | 2 | 5 | 3 | 0.48 |
| Z_2_ | 1/2 | 1 | 3 | 2 | 0.27 |
| Z_3_ | 1/5 | 1/3 | 1 | 1/2 | 0.09 |
| Z_4_ | 1/3 | 1/2 | 2 | 1 | 0.16 |

**Note:** Z_1_ is the economic benefit, Z_2_ is the quality benefit, Z_3_ is water and fertilizer utilization efficiency, and Z_4_ is the environmental benefit.

**Table S5.** Z_1_-Z_11_~Z_12_ judgment matrix

| Index | Z_11_ | Z_12_ | Weights |
| --- | --- | --- | --- |
| Z_11_ | 1 | 1/3 | 0.25 |
| Z_12_ | 3 | 1 | 0.75 |

**Note:** Z_11_ is the output, and Z12 is the net income.

**Table S6.** Z_2_-Z_21_~Z_23_ judgment matrix

| Index | Z_21_ | Z_22_ | Z_23_ | Weights |
| --- | --- | --- | --- | --- |
| Z_21_ | 1 | 7 | 5 | 0.72 |
| Z_22_ | 1/7 | 1 | 1/3 | 0.08 |
| Z_23_ | 1/5 | 3 | 1 | 0.19 |

**Note:** Z_21_ is the starch content, Z_22_ is the vitamin C content, and Z23 is the reducing sugar content.

**Table S7**. Z_3_-Z_31_~Z_32_ judgment matrix

| Index | Z_31_ | Z_32_ | Weights |
| --- | --- | --- | --- |
| Z_31_ | 1 | 1 | 0.5 |
| Z_32_ | 1 | 1 | 0.5 |

**Note:** Z_31_ is water use efficiency, and Z32 is fertilizer utilization efficiency.

**Table S8**. Z_4_-Z_41_~Z_43_ judgment matrix

| Index | Z_41_ | Z_42_ | Z_43_ | Weights |
| --- | --- | --- | --- | --- |
| Z_41_ | 1 | 1 | 1 | 0.33 |
| Z_42_ | 1 | 1 | 1 | 0.33 |
| Z_43_ | 1 | 1 | 1 | 0.33 |

**Note:** Z_41_, Z_42,_ and Z_43_ are for soil nitrate nitrogen, available phosphorus, and available potassium residue, respectively.

**Table S9**.Weights of each evaluation index.

| Criterion | Weights | Indicator layer | Weights |
| --- | --- | --- | --- |
| Z_1_ | 0.48 | Z_11_ | 0.121 |
|  |  | Z_12_ | 0.362 |
| Z_2_ | 0.27 | Z_21_ | 0.197 |
|  |  | Z_22_ | 0.023 |
|  |  | Z_23_ | 0.053 |
| Z_3_ | 0.09 | Z_31_ | 0.044 |
|  |  | Z_32_ | 0.044 |
| Z_4_ | 0.16 | Z_33_ | 0.053 |
|  |  | Z_41_ | 0.053 |
|  |  | Z_42_ | 0.053 |

**Note:** Z_1_ is the economic benefit, Z_11_ is the output, Z_12_ is the net income, Z_2_ is the quality benefit, Z_21_ is the starch content, Z_22_ is the vitamin C content, and Z_23_ is the reducing sugar content. Z_3_ is water and fertilizer utilization efficiency, Z_31_ is water use efficiency, Z_32_ is fertilizer utilization efficiency; Z_4_ for environmental benefit, Z_41_, Z_42,_ and Z_43_ for soil nitrate nitrogen, available phosphorus, and available potassium residue, respectively.

**Table S10**.Objective weights based on the EM.

| Subfactor |  | Z_11_ | Z_12_ | Z_21_ | Z_22_ | Z_23_ | Z_31_ | Z_32_ | Z_41_ | Z_42_ | Z_43_ |
| --- | --- | --- | --- | --- | --- | --- | --- | --- | --- | --- | --- |
| WEM | 2023 | 0.111 | 0.114 | 0107 | 0.106 | 0.100 | 0.114 | 0.111 | 0.106 | 0.074 | 0.056 |
|  | 2024 | 0.114 | 0.114 | 0.100 | 0.103 | 0.099 | 0.108 | 0.115 | 0.065 | 0.097 | 0.086 |

**Note:** Z_11_ is the output, Z_12_ is the net income, Z_21_ is the starch content, Z_22_ is the vitamin C content, and Z_23_ is the reducing sugar content. Z_31_ is water use efficiency, Z_32_ is fertilizer utilization efficiency, and Z_41_, Z_42,_ and Z_43_ are for soil nitrate nitrogen, available phosphorus, and potassium residue, respectively.

**Table** **S11**. Comprehensive weights based on the matrix differential property.

| Subfactor |  | Z_11_ | Z_12_ | Z_21_ | Z_22_ | Z_23_ | Z_31_ | Z_32_ | Z_41_ | Z_42_ | Z_43_ |
| --- | --- | --- | --- | --- | --- | --- | --- | --- | --- | --- | --- |
| W_AE_ | 2023 | 0.121 | 0.349 | 0.192 | 0.028 | 0.056 | 0.048 | 0.048 | 0.056 | 0.054 | 0.053 |
|  | 2024 | 0.121 | 0.348 | 0.191 | 0.028 | 0.056 | 0.048 | 0.048 | 0.054 | 0.056 | 0.055 |

**Note:** Z_11_ is the output, Z_12_ is the net income, Z_21_ is the starch content, Z_22_ is the vitamin C content, and Z_23_ is the reducing sugar content. Z_31_ is water use efficiency, Z_32_ is fertilizer utilization efficiency, and Z_41_, Z_42,_ and Z_43_ are for soil nitrate nitrogen, available phosphorus, and potassium residue, respectively.

**Table S12**. Evaluation criteria of indexes in evaluation index system of potato water and fertilizer coupling effect.

| Indicator | Type | Excellent | Good | Medium | Poor | Worse |
| --- | --- | --- | --- | --- | --- | --- |
| Z_11_ | Positive | 50000 | 45000 | 40000 | 35000 | 30000 |
| Z_12_ | Positive | 50000 | 40000 | 30000 | 20000 | 10000 |
| Z_21_ | Positive | 18 | 17 | 16 | 15 | 14 |
| Z_22_ | Positive | 23 | 22 | 21 | 20 | 19 |
| Z_23_ | Negative | 0.3 | 0.35 | 0.4 | 0.45 | 0.5 |
| Z_31_ | Positive | 15 | 14 | 13 | 12 | 11 |
| Z_32_ | Positive | 60 | 55 | 50 | 45 | 40 |
| Z_41_ | Negative | 50 | 60 | 70 | 80 | 90 |
| Z_42_ | Negative | 40 | 60 | 80 | 100 | 120 |
| Z_43_ | Negative | 400 | 600 | 800 | 1000 | 1200 |

**Note:** Z_11_ is the output, Z_12_ is the net income, Z_21_ is the starch content, Z_22_ is the vitamin C content, and Z_23_ is the reducing sugar content. Z_31_ is water use efficiency, Z_32_ is fertilizer utilization efficiency, and Z_41_, Z_42,_ and Z_43_ are for soil nitrate nitrogen, available phosphorus, and potassium residue, respectively.

**Table S13** Result of a fuzzy comprehensive evaluation

| Years | Treatments | Fuzzy comprehensive evaluation result vector B | | | | | Rating | Score | Rank |
| --- | --- | --- | --- | --- | --- | --- | --- | --- | --- |
|  |  | Excellent | Good | Medium | Poor | Worse |  |  |  |
| 2023 | CK | 0.33 | 0.33 | 0.18 | 0.11 | 0.05 | Excellent | 3.76 | 1 |
|  | C1T1 | 0.21 | 0.28 | 0.38 | 0.06 | 0.06 | Medium | 3.53 | 3 |
|  | C2T1 | 0.17 | 0.11 | 0.11 | 0.31 | 0.30 | Poor | 2.55 | 8 |
|  | C3T1 | 0.21 | 0.21 | 0.13 | 0.06 | 0.38 | Worse | 2.81 | 6 |
|  | C1T2 | 0.23 | 0.35 | 0.31 | 0.05 | 0.05 | Good | 3.65 | 2 |
|  | C2T2 | 0.18 | 0.11 | 0.33 | 0.33 | 0.05 | Medium | 3.05 | 5 |
|  | C3T2 | 0.23 | 0.15 | 0.15 | 0.07 | 0.42 | Worse | 2.71 | 7 |
|  | C1T3 | 0.12 | 0.20 | 0.38 | 0.30 | 0.00 | Medium | 3.15 | 4 |
|  | C2T3 | 0.03 | 0.21 | 0.13 | 0.26 | 0.37 | Worse | 2.26 | 9 |
|  | C3T3 | 0.00 | 0.23 | 0.20 | 0.15 | 0.42 | Worse | 2.25 | 10 |
| 2024 | CK | 0.00 | 0.49 | 0.27 | 0.17 | 0.08 | Good | 3.17 | 10 |
|  | C1T1 | 0.35 | 0.35 | 0.12 | 0.12 | 0.06 | Excellent | 3.82 | 5 |
|  | C2T1 | 0.48 | 0.19 | 0.17 | 0.08 | 0.08 | Excellent | 3.93 | 3 |
|  | C3T1 | 0.28 | 0.39 | 0.14 | 0.14 | 0.06 | Good | 3.69 | 8 |
|  | C1T2 | 0.50 | 0.21 | 0.13 | 0.08 | 0.08 | Excellent | 3.98 | 2 |
|  | C2T2 | 0.55 | 0.19 | 0.09 | 0.09 | 0.09 | Excellent | 4.02 | 1 |
|  | C3T2 | 0.35 | 0.35 | 0.12 | 0.12 | 0.06 | Excellent | 3.82 | 6 |
|  | C1T3 | 0.33 | 0.35 | 0.16 | 0.16 | 0.00 | Excellent | 3.85 | 4 |
|  | C2T3 | 0.30 | 0.38 | 0.16 | 0.13 | 0.03 | Good | 3.79 | 7 |
|  | C3T3 | 0.18 | 0.40 | 0.22 | 0.14 | 0.06 | Good | 3.51 | 9 |

**Note:** CK = control; T1 = biochar produced at 300 °C; T2 = biochar produced at 500 °C; T3 = biochar produced at 700 °C; C1 = 10 t ha^-1^ biochar application; C2=20 t ha^-1^ biochar application; C3=30 t ha^-1^ biochar application.
